# Polyphenolic promiscuity, inflammation-coupled selectivity: Whether PAINs filters mask an antiviral asset

**DOI:** 10.3389/fphar.2022.909945

**Published:** 2022-10-21

**Authors:** Rick Sheridan, Kevin Spelman

**Affiliations:** ^1^ EMSKE Phytochem, Capitola, CA, United States; ^2^ Massachusetts College of Pharmacy and Health Sciences, Boston, MA, United States; ^3^ Health Education and Research, Driggs, ID, United States

**Keywords:** polyphenols, polyphenolic antiviral mechanisms, antiviral MOAs, inflammation, deglucuronidation-through-inflammation mechanism, flavonoids

## Abstract

The Covid-19 pandemic has elicited much laboratory and clinical research attention on vaccines, mAbs, and certain small-molecule antivirals against SARS-CoV-2 infection. By contrast, there has been comparatively little attention on plant-derived compounds, especially those that are understood to be safely ingested at common doses and are frequently consumed in the diet in herbs, spices, fruits and vegetables. Examining plant secondary metabolites, we review recent elucidations into the pharmacological activity of flavonoids and other polyphenolic compounds and also survey their putative frequent-hitter behavior. Polyphenols, like many drugs, are glucuronidated post-ingestion. In an inflammatory milieu such as infection, a reversion back to the active aglycone by the release of β-glucuronidase from neutrophils and macrophages allows cellular entry of the aglycone. In the context of viral infection, virions and intracellular virus particles may be exposed to promiscuous binding by the polyphenol aglycones resulting in viral inhibition. As the mechanism’s scope would apply to the diverse range of virus species that elicit inflammation in infected hosts, we highlight pre-clinical studies of polyphenol aglycones, such as luteolin, isoginkgetin, quercetin, quercetagetin, baicalein, curcumin, fisetin and hesperetin that reduce virion replication spanning multiple distinct virus genera. It is hoped that greater awareness of the potential spatial selectivity of polyphenolic activation to sites of pathogenic infection will spur renewed research and clinical attention for natural products antiviral assaying and trialing over a wide array of infectious viral diseases.

## Introduction

Therapies with demonstrated efficacy for infection by SARS-CoV-2, the etiological agent of the COVID-19 pandemic, include small molecule antivirals such as molnupiravir (Dyer, 2021) and nirmatrelvir (Pfizer, 2021), monoclonal antibodies (U.S.Food and Drugs Administration, 2021), and repurposed drugs such as dexamethasone and fluvoxamine (Reis et al., 2021). Monoclonal antibody therapy suffers from challenging logistics to administer. Dexamethasone has only modest effect on disease outcome (The RECOVERY Collaborative Group, 2021). Fluvoxamine remains prescribable but not yet mandated with agency approvals for COVID-19 (Leo and Erman, 2022). Researchers have called for mutagenicity studies of molnupiravir (Malone and Campbell, 2021; Masyeni et al., 2022). As SARS-CoV-2 variants continue to evolve, nirmatrelvir’s future efficacy could be impacted, including under its own selection pressure on the main protease (Zhou et al., 2022).

Persistently low worldwide vaccination rates, the potential for breakthrough infections, and the ability for vaccinated individuals to achieve viral loads sufficient to infect others (Lipsitch et al., 2021), suggest that there remains ample scope for additional safe, replication-inhibiting antivirals in the panoply of pandemic-alleviating healthcare tools.

Natural products may present a potentially untapped source of antiviral activity. Plants must resist viruses whose constituent peptides are restricted to the same repertoire of proteinogenic amino acids as peptides in mammals. Plant virus proteins share similar fundamental constraints on protein secondary and tertiary structure as viruses with mammalian hosts. Plants’ secondary metabolites are known particularly for plant-protection. Prevalent among the secondary metabolites are polyphenols. One of the three primary polyphenol classes are flavonoids (Quideau et al., 2011).

Flavonoids are a family of over eight thousand unique compounds that provide several advantages to plants (Pietta, 2000; Babu et al., 2009; Terahara, 2015). These compounds are responsible for some pigment and aroma of flowers and fruits, thereby attracting pollinators (Griesbach, 2010; Panche et al., 2016; Mathesius, 2018). Various flavonoids also protect plants from both biotic and abiotic stressors (Takahashi and Ohnishi, 2004; Kumar and Pandey, 2013; Panche et al., 2016), providing antimicrobial defenses (Treutter, 2005; Panche et al., 2016; Mathesius, 2018), acting as UV filters (Sisa et al., 2010; Panche et al., 2016; Mathesius, 2018), and serving as signaling molecules (Mierziak et al., 2014; Panche et al., 2016; Mathesius, 2018). Further, despite sparse literature on the topic, several flavonoids are also demonstrated to inhibit several plant viruses (French and Towers, 1992; Malhotra et al., 1996; Gutha et al., 2010; Likic et al., 2014; Honjo et al., 2020).

Recent research has demonstrated antiviral modes of activity for flavonoids by targeting neuraminidase (Ding et al., 2014; Sharma et al., 2021), proteases (Badshah et al., 2021; Jannat et al., 2021; Sharma et al., 2021), and DNA/RNA polymerases (Badshah et al., 2021). Several flavonoids such as quercetin, apigenin, and luteolin reduce HCV replication through inhibition of multiple viral non-structural proteins (Ninfali et al., 2020). A flavonoid, ladanein inhibited HCV passage into human hepatocytes (Haid et al., 2012). EGCG binds to HSV viral envelope glycoproteins gB and gD, inactivating the virions (Zakaryan et al., 2017). Lalani and Poh (2020)’s survey of flavonoid antiviral studies demonstrated that inhibition of viral enzymes and proteins is the most frequently identified mechanism of action against non-picornaviruses (Lalani and Poh, 2020). Flavonoid compounds from *Sambucus nigra* L. [Adoxaceae] extract were shown to inhibit H1N1 infection by binding to the viral envelope blocking entry into host cells (Roschek et al., 2009). Quercetin demonstrated anti-infective and anti-replicative activity in four different virus species (Middleton, 1998). Quercetin also blocks viral binding and penetration to the host cell in HSV (Zakaryan et al., 2017).

In a recent paper on the antiviral effects of flavonoids, Liskova et al. (2021) review the antiviral mechanisms of action for several flavonoids. For example, caflanone (from *Cannabis sativa* L.-- Cannabaceae) pleiotropically inhibits viral entry factors such as ABL-2, cathepsin L, PI4Kiiiβ and AXL-2, which facilitate mother-to-fetus transmission of coronavirus (Ngwa et al., 2020). In addition, caflanone shows multi-modal anti-inflammatory activity through the inhibition of IL-1β, IL-6, IL-8, TNF-α and Mip-1α (Ngwa et al., 2020). Other flavonoids show anti-inflammatory activity through direct inhibition of NFκB (Rathee et al., 2009). Caflanone, and other flavonoids such as equivir, hesperetin, and myricetin also bind at high affinity to the helicase spike protein of SARS-CoV-2, as well as protease cleavage sites on the ACE2 receptor (Ngwa et al., 2020).

The antiviral effect of *Pelargonium sidoides* DC. [Geraniaceae], also known as umckaloaba, has been found to predominantly depend on the polyphenols, namely the flavonoids and oligomeric proanthocyanidins (Helfer et al., 2014). These compounds have been shown to directly interfere with the infectivity of HIV-1 particles before they interact with the host cell in a polyvalent manner. For instance, the flavonoid/anthocyanidin fraction of *P. sidoides* inhibited attachment of virus particles to cells by inhibiting the early viral proteins of Tat and Rev (positive regulators of gene expression) and inhibited the release of infectious virions. In addition, *P. sidoides* extracts demonstrated a strong reduction of input viral RNA levels in virus-exposed cells. In addition, the previously mentioned flavonoids target HIV-1 envelope proteins (X4 (LAI) and R5 (AD8 and JRFL), thereby inhibiting HIV-1 entry by interfering with the function of the envelope proteins (Helfer et al., 2014).

In *ex-vivo* investigations in rhinovirus-infected cells isolated from patients with severe asthma, moderate COPD, and disease-free controls, a *P. sidioides* extract (standardized to oligomeric prodelphinidins, a type of flavonoid) concentration-dependently demonstrated significantly increased human bronchial epithelial cell survival and decreased expression of inducible co-stimulator (ICOS) and its ligand ICOSL, as well as cell surface calreticulin. In both infected and uninfected, rhinovirus B-defensin-1 and suppressor of cytokine signaling-1 (SOCS1) were up-regulated suggesting a mode of activity for these flavonoid-rich extracts (Roth et al., 2019).

Clinical trials and *in vivo* models of *P. sidoides* flavonoid rich extracts have shown significant efficacy in treating uncomplicated upper respiratory tract infections (URIs) (Gökçe et al., 2021), URIs in asthmatic children (Patiroglu et al., 2012; Tahan and Yaman, 2013), acute bronchitis (Kamin et al., 2010; Kamin et al., 2012), and reduction in bacterial infection *via* immunomodulatory activity (Bao et al., 2015).

Besides the discussed anti-inflammatory activity, there is other immunomodulatory activity of flavonoids which has been reviewed elsewhere (Roshanravan et al., 2020; Liskova et al., 2021; Han et al., 2022). Besides the cytokine inhibition, cytokines have other roles that may significantly affect immune function. For example, the ubiquitous occurring quercetin and its glycoside rutin, have been found to facilitate the shift of macrophages from a proinflammatory to an anti-inflammatory phenotype (Bispo da Silva et al., 2017; Zaragozá et al., 2020). Additionally, apigenin, luteolin, and quercetin show significant immunomodulatory actions on natural killer cell cytotoxicity activity and granule secretion (Oo et al., 2021). Quercetin has also demonstrated a decreased in the expression levels of the major histocompatibility complex class two (MHC II) and costimulatory molecules resulting in a marked reduction of T cell activation (Huang et al., 2010). Finally, human peripheral blood mononuclear cells treated with quercetin preferentially induced interferon gamma (IFN-γ) expression and synthesis while inhibiting IL-4 production resulting in a differential activation of Th1 cells, suggesting potential anti-tumor activity (Nair et al., 2002).

A frequent target of coronavirus antivirals is the SARS-CoV-2 main protease, owing especially to the successful history of protease inhibitors on reducing HIV replication. Several polyphenols showed potent antiviral activity to SARS-CoV’s main protease (Lin et al., 2005; Ryu et al., 2010; Nguyen et al., 2012; Park et al., 2013; Jo et al., 2020). Among these, the polyphenolic flavonoid hesperetin (**1**) was unique in potently inhibiting the action of the main protease in cell-based assay (Lin et al., 2005). Hesperetin dose-dependently inhibited cleavage activity of the 3CLpro in expressed in Vero E6 cells with an IC50 of 8.3 μM (Lin et al., 2005).

**FIGURE 1:**
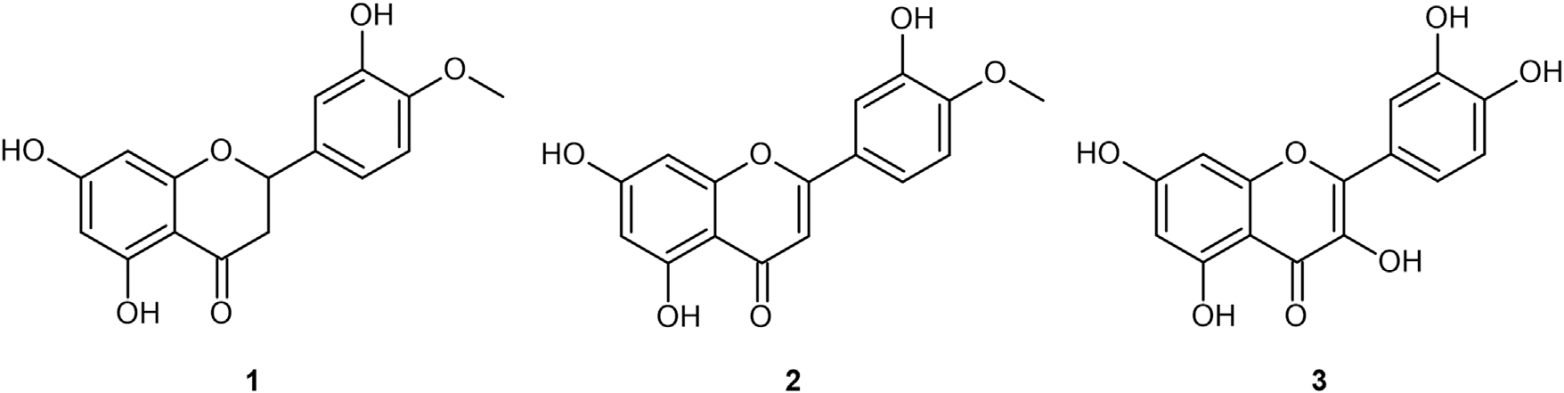
Four flavonoid aglycones referenced in the present work: hesperetin **(1)**; diosmetin **(2)**; quercetin **(3)**.

However, polyphenols like hesperetin are disfavored by industrial medicinal chemists for proceeding through the hit-to-lead (H2L) stage of the drug discovery pipeline (Lowe, 2020; Lowe, 2021). Polyphenols are categorized among the Pan-Assay INterference compounds (PAINs) (Lowe, 2012) [other terms are “frequent-hitters”, “promiscuous inhibitors”, “privileged structures/scaffolds”, and “invalid metabolic panaceas” (Bisson et al., 2016)], and are suggested to obscure the results of various assays. They also bind broadly to assays’ protein targets themselves. Selected examples of polyphenol aglycones are provided in Figure 1.

Due to the ongoing pressing need for further COVID treatment strategies, we review the pharmacokinetic and putative frequent-hitting behavior of polyphenols’ as a class with an eye toward ascertaining 1) their potential as an antiviral 2) whether or not polyphenols simultaneously should pose risks to ordinary healthy cellular processes.

## What defines a polyphenol?

While IUPAC has defined the term “phenols” (Gold, 2019), a definition of polyphenols remains yet to be formally accepted. Quideau (2011) explored definitions of polyphenols extensively, providing an applicable description:

“*The term “polyphenol” should be used to define plant secondary metabolites derived exclusively from the shikimate-derived phenylpropanoid and/or the polyketide pathway(s), featuring more than one phenolic ring and being devoid of any nitrogen-based functional group in their most basic structural expression*.” (Quideau et al., 2011)

Describing polyphenols in part based on their provenance provides excellent exclusivity. However, one could question whether it is helpful to exclude phenols such as acacetin which have only one phenolic ring. For large-scale cheminformatic purposes, which challenge the application of biosynthesis pathway criteria, an applicable definition may be to treat polyphenols as any molecule with more than one phenolic ring but lacking elements other than C, H, and O.

## Poor polyphenol PK perception

The therapeutic efficacy of any antiviral whose purpose is to reduce viral replication requires maintaining an efficacious concentration of the ligand at its putative target for an extended period of time. Conservatively, this period should ideally be of long duration relative to a virus’s replication time to reach peak viral load. An interval typically measured in days in the case of SARS-CoV-2 infection in humans (Kissler et al., 2021).

However, polyphenolic compounds’ potential for efficacy for any particular pathology is criticized due to a prima facie poor pharmacokinetic ADMET profile. Consider, for example, diosmetin (**2**). A primary intermediate metabolite of the pharmaceutical formulation known as Daflon (comprised of 90% diosmin, and 10% other flavonoids expressed as hesperidin, diosmetin, linarin, and isorhoifolin), and similarly proportioned formulations are prescribed in many countries around the world for chronic venous insufficiency (CVI).

**FIGURE 2:**
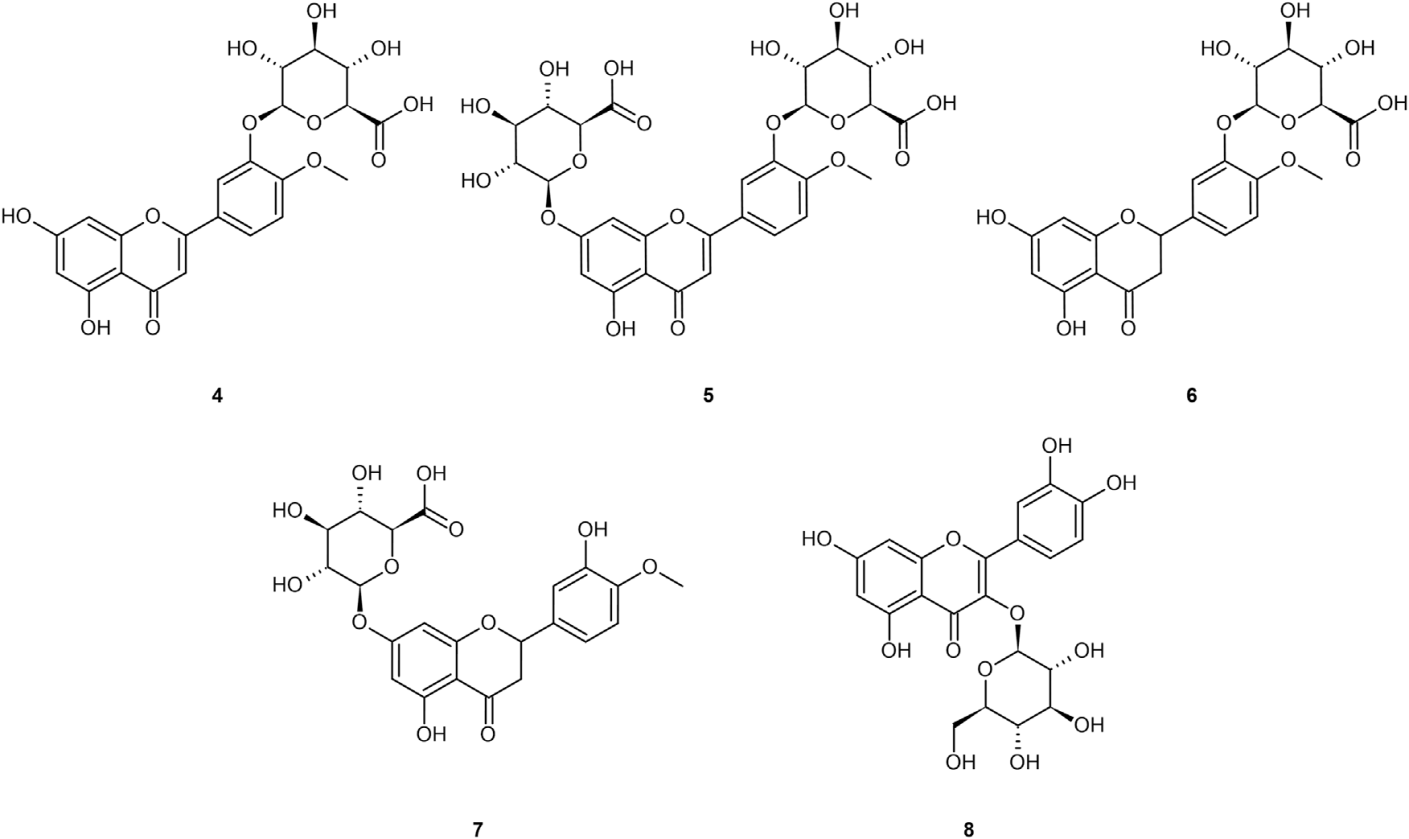
Common glucuronide metabolites of the referenced aglycones: diosmetin-3′-O-glucuronide **(4)**; diosmetin-3′-7-O-glucuronide **(5)**; hesperetin-3′-O-glucuronide **(6)**; hesperetin-7–O-glucuronide **(7)**; quercetin-3-O-glucuronide **(8)**.

Ingested diosmin becomes diosmetin through Phase I metabolism through the intestinal wall, and then is either glucuronidated (primarily) to glucuronides (**4** and **5**), sulfated, or methylated through Phase II metabolism in the liver (Boutin et al., 1993; Meyer, 1994; Struckmann and Nicolaides, 1994; Spanakis et al., 2009; Campanero et al., 2010; Patel et al., 2013; Silvestro et al., 2013; Russo et al., 2015; Russo et al., 2018; Mandal et al., 2019; Bajraktari and Weiss, 2020). Serum analysis on healthy individuals demonstrates negligible presence of the aglycone in plasma, and low sustained levels of the diosmetin conjugates (primarily glucuronides) in plasma, with t_max_ of 2.3 h and t_1/2_ ranging from 8–70 h (Boutin et al., 1993; Struckmann and Nicolaides, 1994; Spanakis et al., 2009; Campanero et al., 2010; Silvestro et al., 2013; Russo et al., 2015; Russo et al., 2018; Mandal et al., 2019; Bajraktari and Weiss, 2020). Stachulski and Meng (2013) and Tranoy-Opalinski et al. (2014) noted that most glucuronides are rapidly eliminated by the kidneys, posing an apparent limitation to their efficacy (Tranoy-Opalinski et al., 2014). Glucuronidation further reduces bioavailability to the intracellular compartment as the glucuronide moiety imparts a hydrophilicity that prevents cellular uptake (Tranoy-Opalinski et al., 2014). Glucuronides of the aglycones from Figure 1 are presented in Figure 2.

**FIGURE 3:**
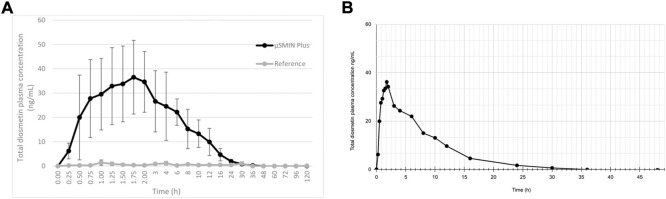
**(A)** Diosmetin plasma concentration (as ascertained following deglucuronidation), after administration of a 50 mg/kg micronized diosmin formulation to rats. Image licensed under CC by 4.0 from Russo et al. (2018) (Russo et al., 2018). **(B)** same plot with linearized axes.

Note that a similar metabolic pathway can be described for other flavonoid aglycones. In the case of hesperidin, it is hydrolyzed to hesperetin, ultimately primarily becoming glucuronides (**6** and **7**) or quercetin, primarily to glucuronide (**8**). Russo et al. (2018) provided a prototypical example of flavonoid plasma pharmacokinetics as demonstrated by diosmetin, which is reproduced and linearized in Figures 3A,B, respectively.

Yet to our knowledge 1) no quantitative bioavailability assays of diosmetin have taken place in non-plasma compartments such as extracellular fluid and tissue in humans; 2) tissue distribution studies of flavonoids in animal models are few. More *in vivo* distribution data to support therapeutic insights into polyphenols would be valuable.

## Drawbacks of polyphenol PK analysis

On broader review of the polyphenol pharmacokinetic literature, five insights about pharmacological assays emerge:

1. The most commonly obtained pharmacological assay for concentration of polyphenols or their metabolites is blood plasma analysis, rather than interstitial fluid or intracellular fluid (Tozer, 1981; Ueno et al., 1983; Boutin et al., 1993; Meyer, 1994; Struckmann and Nicolaides, 1994; Walle et al., 2001; Spanakis et al., 2009; Campanero et al., 2010; Jin et al., 2010; Kaushik et al., 2012; Takumi et al., 2012; Patel et al., 2013; Silvestro et al., 2013; Russo et al., 2015; Yang et al., 2016; Nikiforov, 2017; HealthTech and de Zeneta, 2018; Russo et al., 2018; Mandal et al., 2019; Bajraktari and Weiss, 2020; Hai et al., 2020).
2. A polyphenol’s plasma concentration profile alone provides no data on tissue distribution or biotransformation (Tozer, 1981; Walle et al., 2001; Ratain and Plunkett, 2003).
3. It is very difficult to sample intracellular fluid for drug/metabolite concentration profiling to the exclusion of extracellular and serum fluid (Lowe, 2018; Lowe, 2019).
4. Even radiolabeled assaying of all possible elimination routes fails to provide a complete accounting of polyphenol dosage intake (Walle et al., 2001).
5. Plasma samples of polyphenols are more frequently obtained from healthy individuals, rather than those suffering from a particular pathology (Ueno et al., 1983; Boutin et al., 1993; Meyer, 1994; Struckmann and Nicolaides, 1994; Walle et al., 2001; Spanakis et al., 2009; Campanero et al., 2010; Jin et al., 2010; Kaushik et al., 2012; Takumi et al., 2012; Patel et al., 2013; Silvestro et al., 2013; Russo et al., 2015; Yang et al., 2016; Nikiforov, 2017; HealthTech and de Zeneta, 2018; Russo et al., 2018; Mandal et al., 2019; Bajraktari and Weiss, 2020; Hai et al., 2020).

Therefore, if any particular pharmaceutical candidate’s PK profile achieves significant distribution in organs other than those associated with either the GI tract or renal tract, it would be unascertainable from serum analysis alone. Further, if any particular pathology has an effect on a compound’s tissue distribution (whether by causing sequestering in sanctuary sites, or adduct formation with the target in tissue both of which represent an increase in the volume of distribution), then plasma analysis alone remains poorly positioned to provide the relevant readout. Rather, tissue analysis in sacrificed animal models, or comprehensive radiolabeled elimination quantitation in humans, would be required.

Walle et al. (2001) demonstrated such a radiolabeled analysis. Notably, they found that carbon dioxide was a major metabolite of quercetin (**3**) in humans, (Walle et al., 2001) suggesting a rarer elimination pathway than typically encountered by pharmacological analysis. Even with this exotic elimination route taken into account, the full dose of quercetin was not always fully representative of the dose given. One can speculate that sequestration of quercetin products in tissue compartments was maintained past the 72-hr study period.

Moreover, while de Boer et al. (2005) and Bieger et al. (2008) demonstrated that quercetin reaches certain tissues other than those associated with GI and renal tracts in healthy animal models, these studies do not address any putative bioavailability of flavonoids uniquely to tissue affected by diseased circumstances.

## Toward resolving the “flavonoid paradox”

To begin addressing these pharmacokinetic challenges, we examine them through the lens of a subtle but critically important feature of the pharmacokinetic profile of polyphenols, such as flavonoids, as elucidated by the literature.

Menendez et al. and Perez-Vicaino et al. frame flavonoids’ pharmacokinetic challenges in the context of a “flavonoid paradox” (Menendez et al., 2011; Perez-Vizcaino et al., 2012). The paradox can be summarized as the observation that several flavonoid polyphenols have been shown to demonstrate therapeutic effects for various pathologies *in vivo*, and yet their pharmacological profiles suggest poor bioavailability, including the difficulty of glucuronide metabolites to pass through cell membranes, along with rapid plasma clearance.

Investigators (Marshall et al., 1988; Shimoi et al., 2000; O’Leary et al., 2001; Shimoi et al., 2001; Shimoi and Nakayama, 2005; Kawai et al., 2008; Bartholomé et al., 2010; Menendez et al., 2011; Galindo et al., 2012; Ishisaka et al., 2013; Kaneko et al., 2017; Piwowarski et al., 2017; Ávila-Gálvez et al., 2019) of the following deconjugation mechanism offer a resolution to the flavonoid paradox: During inflammation (as happens during infection of several etiologies), phagocytes arrive at the extracellular fluid surrounding the sites of inflammation. The phagocytes express β-glucuronidase which accomplishes deglucuronidation (also known as deconjugation) of the flavonoid glucuronide into its aglycone form. The deconjugated flavonoid aglycone subsequently diffuses through the cell membrane where they can reach their target. The mechanism is summarized in Table 1. For purposes of this review, the mechanism steps are labeled stages B, C, D, E, and F.

**TABLE 1:** Deglucuronidation-through-inflammation mechanism steps.

Stage B—Flavonoid aglycones are glucuronidated prior to arrival in the bloodstream
↓
Stage C—Neutrophils and macrophages are attracted to site(s) of inflammation
**↓**
Stage D—β-glucuronidase is expressed by neutrophils and macrophages
**↓**
Stage E—Serum flavonoid glucuronides are deglucuronidated (‘deconjugated’) by β-glucuronidase at site of inflammation
**↓**
Stage F—Flavonoid aglycones diffuse through cell membrane

Comparable pathology-specific activation of glucuronide drugs by β-glucuronidase has been examined and exploited in the context of anti-tumor agents (Tranoy-Opalinski et al., 2014). However, it is the “deconjugation in inflammation hypothesis” that was developed and supported progressively over the period 2000–2019 across several polyphenols *in vitro*, in animal models, and in humans that describes flavonoid conversion to cell-penetrating forms uniquely under inflammatory conditions (Marshall et al., 1988; Shimoi et al., 2000; O’Leary et al., 2001; Shimoi et al., 2001; Shimoi and Nakayama, 2005; Kawai et al., 2008; Bartholomé et al., 2010; Menendez et al., 2011; Galindo et al., 2012; Ishisaka et al., 2013; Kaneko et al., 2017; Piwowarski et al., 2017; Ávila-Gálvez et al., 2019).

**FIGURE 4:**
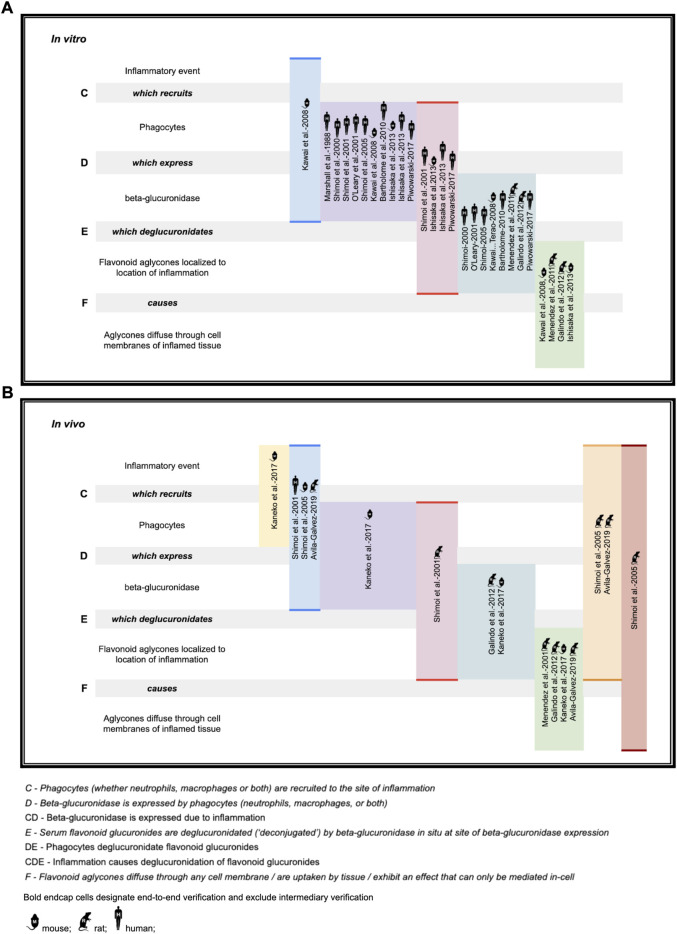
Deconjugation-through-inflammation literature basis **(A)** *in vitro* support **(B)** *in vivo* support.

While the deglucuronidation-through-inflammation hypothesis has been reviewed extensively by others, (Terao et al., 2011; Perez-Vizcaino et al., 2012; Kawai, 2014; Kawabata et al., 2015; Terao, 2017; Kawai, 2018) to our knowledge, this review is the first to unify the body of work into one cohesive, accessible evidentiary framework. Demonstration of the evidence generated through the deglucuronidation-through-inflammation body of work, (Marshall et al., 1988; Shimoi et al., 2000; O’Leary et al., 2001; Shimoi et al., 2001; Shimoi and Nakayama, 2005; Kawai et al., 2008; Bartholomé et al., 2010; Menendez et al., 2011; Galindo et al., 2012; Ishisaka et al., 2013; Kaneko et al., 2017; Piwowarski et al., 2017; Ávila-Gálvez et al., 2019) is provided against the model’s labeled stages C-F in Figure 4.

Figure 4 illustrates the body of deglucuronidation-through-inflammation literature thusly: Original research investigators produced results demonstrating any one of the four stages of the mechanism (which we refer to here as steps C, D, E, F), the entire mechanism (CDEF), or consecutive combinations of steps (such as CD, CDE). We highlight whether evidence for an individual step has been produced, or instead over multiple consecutive steps end-to-end (without isolated verification of any intermediate step). Consecutive step verifications are illustrated by highlighting in bold the top and bottom of the relevant cells.

The figure annotates whether experiments have been performed *in vitro* (using mice, rat, or human cell lines) or *in vivo* in mice models, rat models, or humans. While steps of the pathway were verified across the polyphenols luteolin (Shimoi et al., 2000; Shimoi et al., 2001; Shimoi and Nakayama, 2005), quercetin (O’Leary et al., 2001; Bartholomé et al., 2010; Menendez et al., 2011; Galindo et al., 2012; Ishisaka et al., 2013; Kawai, 2014), daidzein (O’Leary et al., 2001), and kaempferol (O’Leary et al., 2001), as well as the ellagic acid metabolites urolithin A (Piwowarski et al., 2017; Ávila-Gálvez et al., 2019; Bobowska et al., 2021), iso-urolithin A (Piwowarski et al., 2017; Bobowska et al., 2021), and the single-phenol urolithin B (Piwowarski et al., 2017; Bobowska et al., 2021), these are not annotated in the figure for brevity.

We believe the figure also adds value as it makes clear which steps are already demonstrated so that they can undergo simpler replication studies, as well as identifying which steps, such as *in vivo* human verification work at stages DEF, could be better elucidated with fresh original research.

We propose standardizing the mechanism’s naming to the technical term “deglucuronidation-through-inflammation” or DTI. The term ‘Shimoi pathway’ further serves as a convenient shorthand that recognizes the lead researcher to first propose and study this mechanism with specific attention to inflammation with polyphenols.

## The promiscuous inhibition of polyphenols

Promiscuous inhibition poses two primary implications for medicinal chemistry assaying. The first is the non-specific binding of protein/enzyme targets themselves. The second is the disruption of assay integrity by inhibiting non-target enzymes used for assay readout. As it can be difficult to distinguish between these two, orthogonal assays are sometimes performed to verify a target binding interaction.

**FIGURE 5:**
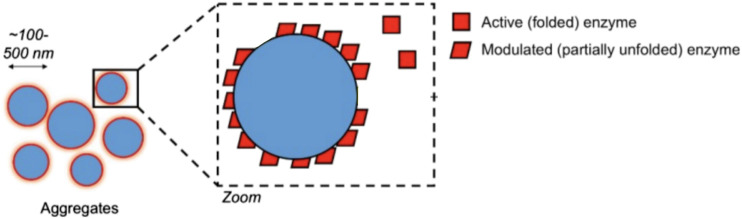
Non-selective aggregation inhibition model. Figure modified from Auld et al. (2017) for clarity under CC BY-NC-SA 3.0.

Promiscuity could take any of several forms. A promiscuous ligand could simply be highly conforming to a protein surface’s geometry, with a high number of hydrogen donors & acceptors to more likely “stick” nonspecifically to any given protein site’s own set of H-donors and H-acceptors. Another mechanism sees promiscuous inhibition take the form of colloidal aggregations (Shoichet, 2006). In this mechanism, upon reaching a certain concentration, the ligand forms tightly-packed spherical aggregates with itself, even inside the cell (Ganesh et al., 2017; Shoichet, 2021) as illustrated in Figure 5. Proteins and enzymes non-specifically bind to the surface of the aggregation and are inhibited in the process (Auld et al., 2017). Often seen as a nuisance originally, it is now also seen as a source of opportunity in drug discovery as well (Ganesh et al., 2017; Ganesh et al., 2018; Ganesh et al., 2019). Deliberate study of aggregation in cell-based assays is a nascent sub-field, (Owen et al., 2012) thus cataloguing of non-specific aggregation among polyphenols in cells merits further investigation.

Quercetin has earned a reputation as a promiscuous inhibitor (Pohjala and Tammela, 2012; Jasial et al., 2016a; Gilberg et al., 2019; Lowe, 2020) as well as having served as one of the first aggregators identified. (McGovern et al., 2002; McGovern and Shoichet, 2003; Shoichet, 2006). Luteolin (Jasial et al., 2016a), curcumin (Jasial et al., 2016a), myricetin (Pohjala and Tammela, 2012; Jasial et al., 2016a; Jasial et al., 2016b; Gilberg et al., 2018; O’Donnell et al., 2021), and tannic acid (Pohjala and Tammela, 2012; Jasial et al., 2016a) are also promiscuous inhibitors, where myricetin and tannic acid have been further identified as aggregators (Pohjala and Tammela, 2012).

Of 123,844 assay records hosted by Pubchem and compiled by Gilberg et al. (2016) (Gilberg et al., 2016), their isolation of the most promiscuous 466 of them (99.6% percentile) contains 13 polyphenols based on our cheminformatic-oriented definition.

The catechol functional group, while not the exclusive province of polyphenols (and nor do polyphenols all contain catechol), certainly correlates with polyphenols. Bael and Holloway (2010), highlights catechol as a prominent PAINS functional group (Baell and Holloway, 2010) even as Capuzzi et al. (2017) cautions against blind application of PAINS filters. (Capuzzi et al., 2017). And yet Jasial et al. (2017) demonstrates that the catechol functional group is in the top ten percentile (9.5) of primary activity assays in Pubchem, and in the top seven percentile (6.9) of functional groups in Pubchem confirmatory assay activity (Jasial et al., 2017).

## Therapeutic role of a promiscuous binder?

The final step of a putative polyphenol deglucuronidation-based antiviral mechanism requires that a promiscuous-binding compound once inside a virus-infected human cell will arrest viable virion production. The complete proposed mechanism is presented in Table 2.

**TABLE 2:** Proposed deglucuronidation-based antiviral mechanism.

**Stage A—Infection by any of several virus species induces inflammation**
↓
Stage B—Flavonoid aglycones are glucuronidated prior to arrival in the bloodstream
↓
Stage C—Neutrophils and macrophages are attracted to site(s) of inflammation
↓
Stage D—β-glucuronidase is expressed by neutrophils and macrophages
↓
Stage E—Serum flavonoid glucuronides are deglucuronidated (“deconjugated”) by β-glucuronidase at site of inflammation
↓
Stage F—Flavonoid aglycones diffuse through cell membrane
↓
**Stage G—Flavonoid aglycones cause non-selective (and non-specific) inhibition within the cell—interfering with both ordinary cellular processes and the etiological source of inflammation (such as viral replication)**

**FIGURE 6:**
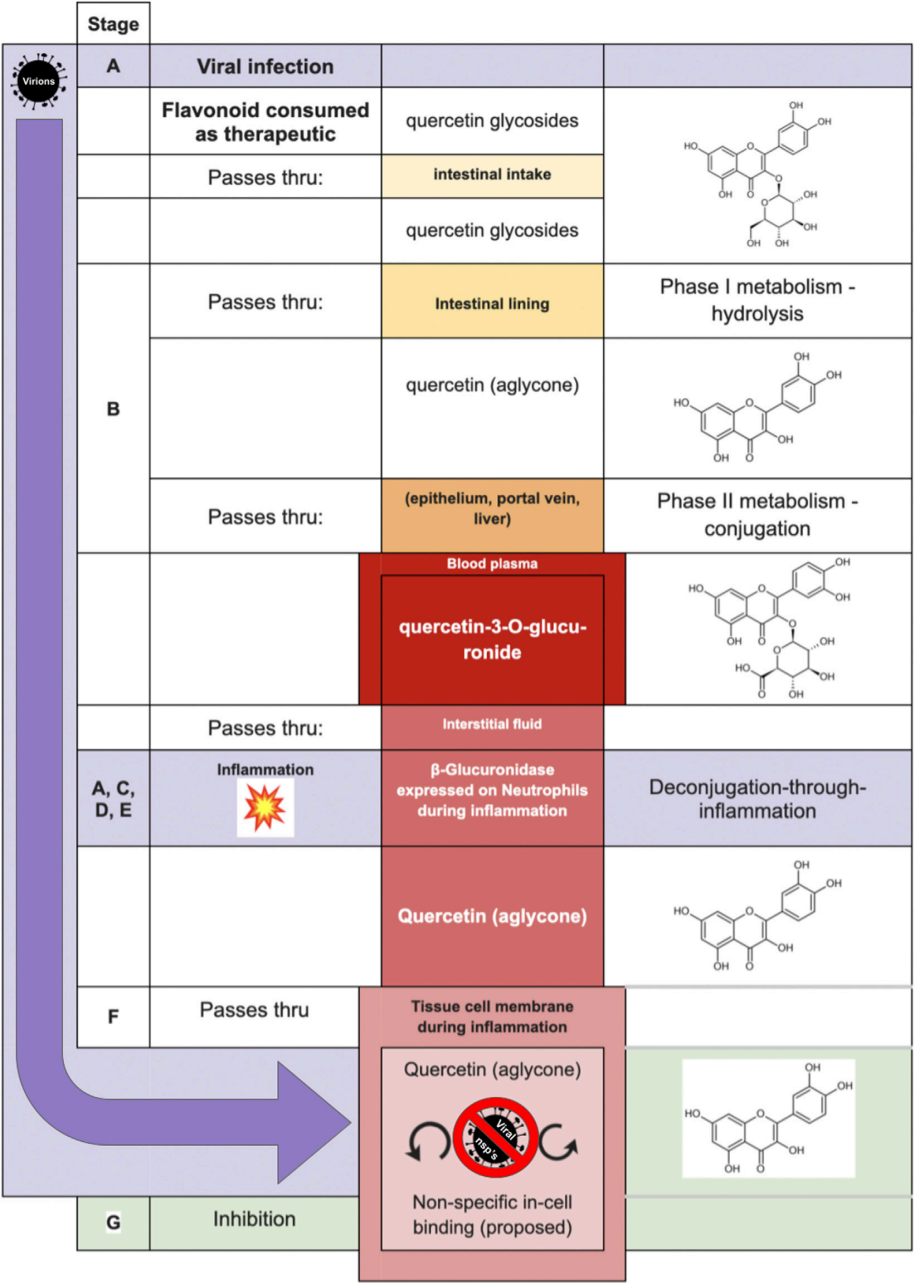
Proposed model of Shimoi mechanism for entry into intracellular compartment during viral infection, with quercetin serving in the role of the aglycone, and quercetin-3-O-glucuronide as the glucuronide [adapted from Perez-Vizcaino et al. (2012)].

Table 2 is illustrated graphically in Figure 6—by way of one of the most studied flavonoids in the pharmacokinetic literature, quercetin. Inhibitory mechanisms of viral replication could be due to direct inhibition of viral proteins and enzymes, or by slowing ordinary cellular metabolic mechanisms such as respiration, translation, transcription as co-opted by the infecting virus. In one case, that of fisetin applied to Dengue fever, (Zandi et al., 2011a) fisetin showed no direct activity against DENV virions outside the cell yet effectively inhibited replication in-cell. The study’s authors suggest it could be due to forming complexes with RNA or inhibition of RNA polymerases. While inhibition of the dengue RdRp would represent a virus-specific inhibition, it remains intriguing to consider that the replication inhibition could also be due to general non-selective inhibition in a weakened cell.

## Application of deglucuronidation-through-inflammation to antiviral assaying and clinical trialing

Given that many forms of viral infections are known to induce inflammation, it would be a logical extension to study whether consumption of certain flavonoids of sufficient quantity and in bioavailable forms could serve to reduce the rate of viral replication in the early stages of viral infection. The mechanism of action could be by inhibition of viral entry to cells, direct inhibitory action on viral enzymes in-cell, or non-specific promiscuous disruption of the co-opted metabolism of infected cells.

**FIGURE 7:**
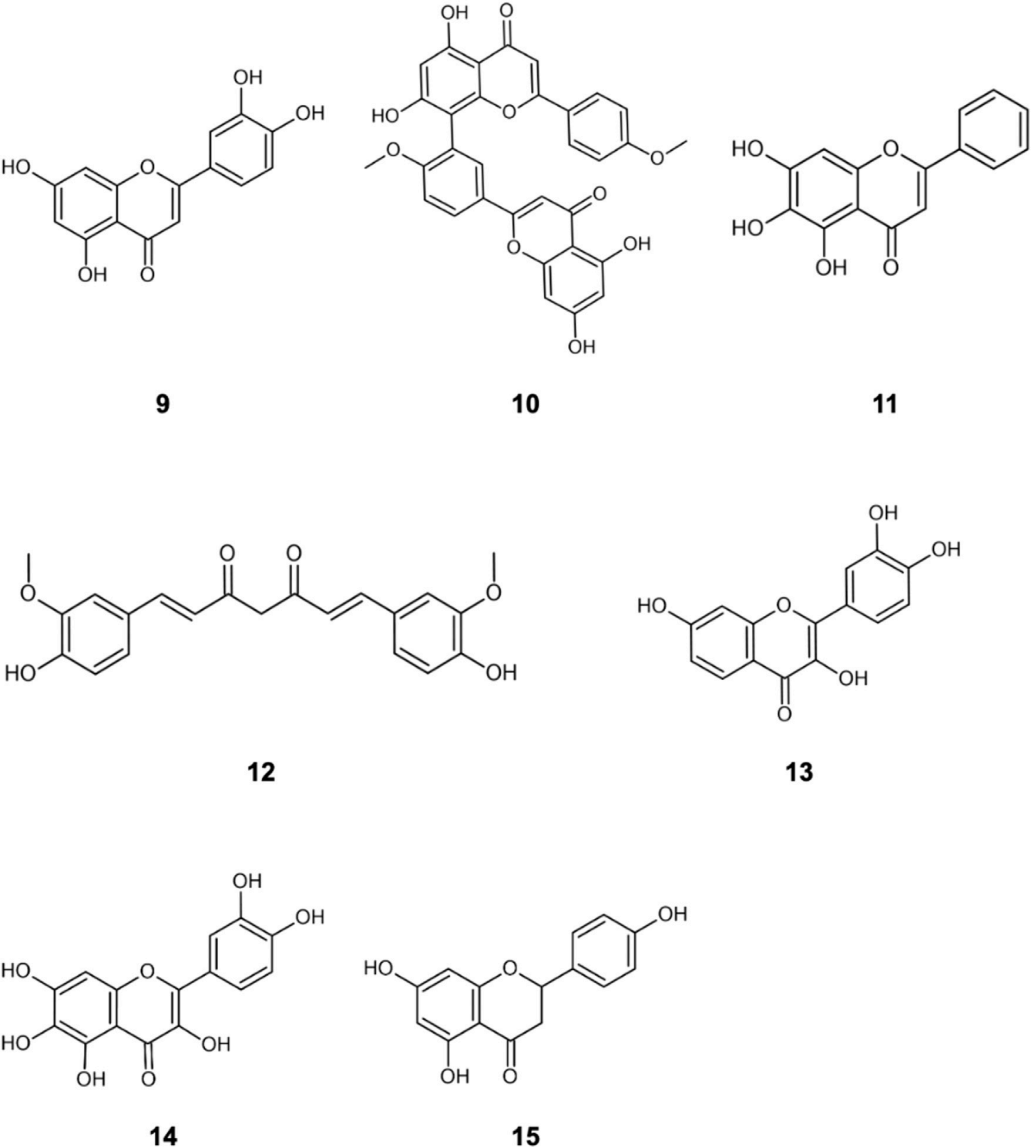
Additionally referenced polyphenols: luteolin **(9)**; Isoginkgetin **(10)**; baicalein **(11)**; curcumin **(12)**; fisetin **(13)**; quercetagetin **(14);** naringenin **(15)**.

The literature provides early *in vitro* evidence of achievable inhibition by phenolic flavonoids spanning across Dengue virus, Influenza-A virus (IAV), Chikungunya virus, Foot-and-mouth disease virus (FMDV), Japaneses Encephalitus Virus (JEV) and SARS-CoV-2, presented in Table 3. Corresponding ligand structures are available in Figure 1 and Figure 7.

**TABLE 3:** *in vitro* evidence of in-cell viral inhibition (reported IC50) by phenols and polyphenols.

	DENV	FMDV	Influenza-A	JEV	CHIKV	ZIKV	SARS-CoV-2
**Luteolin** **(9)**		**9.7–10.0 μM** Natural phytochemicals (2002)	**6.9–7.2 μM** Yan et al. (2019)	**15.9 μM** Fan et al. (2016)			
**Isoginkgetin** **(10)**		**1.9–2.0 μM** Natural phytochemicals (2002)					
**Quercetin** **(3)**	**95.6–118 μM** Zandi et al. (2011b)		**8.9–25.8 μM** Wu et al. (2015)			**2.3 μM** Zou et al. (2020)	**18.2 μM** Kandeil et al. (2021)
**Baicalein** ^a^ **(11)**	**5.7–23.9 μM** Zandi et al. (2012)			**12.1–52.8 μM** Johari et al. (2012)	**7.0 μM** Lani et al. (2016)		**2.9 μM** Liu et al. (2021)
**Curcumin** **(12)**	**14.0 μM** Balasubramanian et al. (2019)		**0.5–3.8 μM** Kim et al., (2021), Chen et al. (2010)	**<30 μM** Chen et al. (2010)	**3.9 μM ** Mounce et al. (2017)	**<1.9 μM** Mounce et al. (2017)	**0.4–38 μM** Bormann et al. (2021), Kandeil et al. (2021), Marín-Palma et al. (2021)
**Fisetin** **(13)**	**150 μM** Zandi et al. (2011a)				**29.5 μM** Lani et al. (2016)		
**Quercetagetin** **(14)**					**43.5 μM** Lani et al. (2016)		
**Hesperetin** **(1)**					**8.5 μM** Ahmadi et al. (2016)		
**Naringenin** **(15)**	**18–180 μM** Zandi et al. (2011a)				**6.8 μM** Ahmadi et al. (2016)		**<35 μM** Clementi et al. (2021)

^a^ One phenol only.

Much care must be applied in interpreting *in vitro* viral replication inhibition results. Where an IC50 value is defined against a measure of viral RNA copies/mL, then qRT-PCR will show a difference of a single unit of Ct. For comparison, SARS-CoV-2 infection typically presents a Ct range between 10 and 40 for acute infection vs. non-detectable viral load, respectively (Kissler et al., 2021). However, *in vitro* and *in vivo* Ct values are not directly comparable, as *in vitro* reduction of viral replication may exhibit nonlinear effects at the *in vivo* scale, especially when the effects of the innate and specific immune system are considered. Where due analysis of toxicity allows, a higher IC value can be targeted, such as IC90 or even IC99 (Rusconi et al., 1994).

Viral inhibitory assays typically report the Selectivity Index (SI), defined as the ratio of the cytotoxicity (CC50) to the inhibitory concentration (IC50). A SI < 1 means that the ligand’s cytotoxicity to cells occurs at a lower concentration than its inhibition of the target. Selectivity indices of 5 or greater are preferred. Ligand candidates suffering from lower selectivity indices may be excluded from further investigation. However, the deglucuronidation-through-inflammation mechanism would suggest that dismissing polyphenol ligands with a low selectivity index could be overly conservative. Given that polyphenols circulate in plasma primarily as glucuronides, (Boutin et al., 1993; Shimoi et al., 1998; Kuhnle et al., 2000; Shimoi et al., 2000; O’Leary et al., 2001; Manach et al., 2003; Campanero et al., 2010; Silvestro et al., 2013; Russo et al., 2015; Russo et al., 2018; Bajraktari and Weiss, 2020; Hai et al., 2020) a low selectivity index for the aglycone may not only be acceptable but may even be preferable. This of course will depend on how efficiently the deglucuronidation process discriminates between the localities of healthy and infected cells that induce the inflammatory process.

Given the selectivity that deglucuronidation-through-inflammation affords, and the putative validity of aggregation-based non-selective binding mechanisms, (Shoichet, 2021) the standard practice of applying aggregate-dissociating detergents such as Triton X-100 is called into question for antiviral assays of phenols that are known or expected to act through the DTI mechanism. A revisiting of relevant results of *in vitro* assays in the literature where such a detergent was applied would be appropriate. However, such a modification to laboratory practice should be considered carefully as the tendency for an aggregation to bind assay-specific enzymes could still benefit from detergent application.

Non-selective inhibition can be a double-edged sword. Dong (2014) demonstrates that the aglycone kaempferol increased IAV viral titers by log-2 compared with untreated mice, hastening their loss (Dong et al., 2014). This was attributed to attenuation of antiviral host-defense factor expression such as IFNα, IFNβ, IFNγ. By contrast, hesperidin was protective of the mice. Further laboratory and clinical investigation of demonstrably promiscuous-binding polyphenols utilizing *in vitro* viral infection culture and *in vivo* models will continue to be valuable. Attention would be particularly appropriate against those viruses that are known to induce inflammatory responses such as influenza A (IAV-A), dengue (DENV), chikungunya (CHIKV), and coronavirus (SARS-CoV-2).

## Additional observations on antivirals trialing of polyphenols

While it is important to maintain ligand concentration at the target for a period sufficient to exert the relevant mechanism of action, it is worth noting that this period can be extremely short. Although not a polyphenol, artemisinin enjoys enormous efficacy against the malaria parasite *Plasmodium falciparum* with a T_max_ at less than 2 h and a half-life of 2–5 h (Benakis et al., 1997; de Vries and Dien, 2021). Also, the dosage is of utmost importance. Following due analysis of toxicity, protease inhibitors can target a C_min_ dose (minimum concentration between consecutive doses) of many multiples of the IC50 value (Boras et al., 2021) to achieve faster viral clearance. Indirect antiviral effects of certain polyphenols may also be possible, such as non-specific upregulation of immunosurveillance, as well as modulation of specific immune cells (Liu et al., 2016; Kang et al., 2021; Syafni et al., 2021).

Also, as promiscuous binders, due attention should be applied to inhibition of liver enzymes for drug-drug interactions (Bajraktari and Weiss, 2020), especially of drugs that study subjects might concomitantly consume for the same or unrelated conditions. For example, among the polyphenols studied are those known to bind to CYP1A2 (Bajraktari and Weiss, 2020), CYP3A4 (Bajraktari and Weiss, 2020) and OATP1A2, the latter giving rise to the famous “grapefruit effect” (Bailey et al., 2007). Conversely, this P450 or other liver enzyme inhibition may be advantageous to increase serum concentrations of verified pharmaceuticals, such as fluvoxamine, in context of combination therapy (James Duke, personal communication, 28 November 2009) for superior joint bioavailability.

Finally, as a given polyphenol can demonstrate differing bioavailabilities between different dosage forms, (Kaushik et al., 2012) consideration should also be given to oral delivery type, such as aqueous, softgel, dry tablet form, and degree of micronization. Further, owing to strong bioavailability and/or release rate, delivery in the form of original plant matter while controlling for phytochemical content should also be considered (Kawai et al., 2008; Inoue et al., 2015; Hu et al., 2017).

## Evolutionary role

A BLAST search demonstrates that the gene coding for β-glucuronidase [GUSB, and uidA in bacteria (Martins et al., 1993)] is extensively common across the animal kingdom (data not shown). Its homolog β-galactosidase (40% identity) is also commonly expressed in bacteria (data not shown). β-glucuronidase has several documented purposes (Martins et al., 1993). It targets glucuronic acid in the gut, (Wallace et al., 2015; Dashnyam et al., 2018) and is associated with the degradation of glucuronate-containing glycosaminoglycan (Naz et al., 2013). But its extensive expression on, and release from, neutrophils attracted in response to inflammatory signals is a mechanism whose genetic etiology and species prevalence will require further work to elucidate.

Given the long history of herbivory in animals (and associated polyphenolic compound ingestion), and the high prevalence frequency of the β-glucuronidase-coding gene GUSB across vertebrates, this mechanism could be a long-ago evolved broad response under selection pressure of viral pathologies in ancestral species. It would be worthwhile for future investigators to probe the genetic basis for neutrophilic β-glucuronidase expression and its orthology across vertebrate species in order to better localize how this response evolved.

## Conclusion

In this paper, we add to the body of evidence in the literature that polyphenols are a frequent-binding class of chemicals produced by plants. We show that the pharmacology of polyphenols may allow for viral infection-fighting potential due to the human body’s inflammatory response and provide conjecture as to the evolutionary basis for a putative inflammation-induced antiviral function. Future work could include quantifying the effect of *in vitro* antiviral studies under inflammation with neutrophils present for such viral targets as SARS-CoV-2, CHIKV, DENV, and IAV/IBV.
